# Weight Loss Trajectories and Related Factors in a 16-Week Mobile Obesity Intervention Program: Retrospective Observational Study

**DOI:** 10.2196/29380

**Published:** 2022-04-15

**Authors:** Ho Heon Kim, Youngin Kim, Andreas Michaelides, Yu Rang Park

**Affiliations:** 1 Department of Biomedical Systems Informatics Yonsei University College of Medicine Seoul Republic of Korea; 2 Noom Inc New York, NY United States

**Keywords:** clustering, mobile health, weight loss, weight management, behavior management, time series analysis, mHealth, obesity, outcomes, machine learning, mobile app, adherence, prediction, mobile phone

## Abstract

**Background:**

In obesity management, whether patients lose ≥5% of their initial weight is a critical factor in clinical outcomes. However, evaluations that take only this approach are unable to identify and distinguish between individuals whose weight changes vary and those who steadily lose weight. Evaluation of weight loss considering the volatility of weight changes through a mobile-based intervention for obesity can facilitate understanding of an individual’s behavior and weight changes from a longitudinal perspective.

**Objective:**

The aim of this study is to use a machine learning approach to examine weight loss trajectories and explore factors related to behavioral and app use characteristics that induce weight loss.

**Methods:**

We used the lifelog data of 13,140 individuals enrolled in a 16-week obesity management program on the health care app Noom in the United States from August 8, 2013, to August 8, 2019. We performed k-means clustering with dynamic time warping to cluster the weight loss time series and inspected the quality of clusters with the total sum of distance within the clusters. To identify use factors determining clustering assignment, we longitudinally compared weekly use statistics with effect size on a weekly basis.

**Results:**

The initial average BMI value for the participants was 33.6 (SD 5.9) kg/m^2^, and it ultimately reached 31.6 (SD 5.7) kg/m^2^. Using the weight log data, we identified five clusters: cluster 1 (sharp decrease) showed the highest proportion of participants who reduced their weight by >5% (7296/11,295, 64.59%), followed by cluster 2 (moderate decrease). In each comparison between clusters 1 and 3 (yo-yo) and clusters 2 and 3, although the effect size of the difference in average meal record adherence and average weight record adherence was not significant in the first week, it peaked within the initial 8 weeks (Cohen *d*>0.35) and decreased after that.

**Conclusions:**

Using a machine learning approach and clustering shape-based time series similarities, we identified 5 weight loss trajectories in a mobile weight management app. Overall adherence and early adherence related to self-monitoring emerged as potential predictors of these trajectories.

## Introduction

### Background

The worldwide prevalence of overweight or obesity has doubled since 1980 [1]. In the United States, the prevalence of obesity has increased dramatically among both adults and children [2]. Meanwhile, epidemiologic research has identified high BMI values as a risk factor for an expanding set of chronic diseases, including cardiovascular disease, diabetes mellitus, kidney disease, and several cancers [1].

There is a need for effective obesity interventions that can reach large population sectors at low cost. With 66% of the world’s population owning tablets or smartphones [3], web-based interventions can facilitate the implementation of wide-reaching, self-directed approaches to tackle obesity. Self-directed interventions require minimal contact with professionals and empower participants to control and regulate their thoughts themselves [4]. Relevant guidelines and systematic reviews have widely recognized self-directed interventions as effective intervention techniques for obesity treatment [5]. Several studies have examined the effectiveness of self-directed interventions [5-10] and have suggested that such approaches could be used to deliver improved and personalized care while reducing health care costs [5].

### Weight Loss and Its Predictors

Individuals who lose 5%-10% of their initial weight are generally considered as responding to their obesity treatment: these proportions are clinically associated with improvement in cardiovascular risk factors [11]. In several weight loss intervention trials, participants who lost 5%-10% of their initial weight by the end were considered responders [12-14]. However, this approach does not distinguish between individuals with widely varying weight changes and those who steadily lose weight throughout the intervention period. Furthermore, little is known about patterns of individual week-to-week weight changes and about how these changes may relate to weight loss achievement (or a lack thereof) [14]. Such evidence could shed light on the relationship between weight loss and its predictors from a longitudinal perspective and would be of potential use in tailoring weight management and behavior change interventions, especially for groups at risk of suboptimal outcomes [15].

Research has yet to outline weight loss trajectories in individuals who have participated in self-directed interventions using mobile phones. Therefore, this study aims to (1) identify weight loss trajectories and (2) explore factors related to behavioral and health care app use characteristics eliciting weight loss.

## Methods

### Study Design and Participants

In this retrospective study, we obtained deidentified user log data from Noom, Inc, which provides mobile app services. Noom is a mobile-based wellness app that focuses on behavior changes to enable weight loss. Through this app, users can receive personalized and one-on-one coaching from health experts and self-monitor their food intake and exercise. Coaching encourages users to record their weight on a weekly basis and meals on a daily basis. In addition, this app enables users to record their energy intake with higher reliability than professional software [16]. Users select the volume of their intake or units from which calorie intake can be measured. By spurring users to record their meals, exercise sessions, and weight, this app keeps them aware of their weight status and dietary patterns. This program was modeled after the Diabetes Prevention Program, sponsored by the National Institute of Diabetes and Digestive and Kidney Diseases, where participants received 16 weeks’ worth of content over the first 6 months. There is additional curriculum past the 16-week mark to support users for approximately 1 year.

The retrospective cohort for this study included 93,814 users in the United States who were enrolled in the coaching program provided by Noom from August 8, 2013, to August 8, 2019. To target users with the same intervention duration, we only included those users who participated in the program for 16 weeks. Among these, we excluded app users without weight records because we could not evaluate their outcomes. We also excluded users with a height <125 cm or >230 cm, which is a loosened criterion of US Census protocol and these cutoff points correspond to 0.01% or 99.99%, respectively, of height distribution of our cohort [17]. Next, we excluded users with age data not meeting the inclusion criteria, such as young users or those who did not enter their age when signing up. To focus on individuals who needed to manage their weight, we included adult users with a BMI higher than the criterion for overweight (≥25 kg/m^2^), which could potentially pose health problems such as cardiovascular disease based on the recommendations of the Centers for Disease Control and Prevention [18]. In addition, because this app (Noom) can include users who are underweight and want to gain weight from dietary management, this allowed us to focus on individuals who would be classified as overweight. Furthermore, we selected users with weight records available during the last week of the program or between the end of the program and 1 week after the end of the program to identify the final outcome of the weight loss program. Finally, we excluded users with inconsistent weight records that showed a difference in BMI of ≥3.5 kg/m^2^ between consecutive time points within 1 month or of ≥7.0 kg/m^2^ between consecutive time points within 2 months [19].

### Data Acquisition and Preprocessing

We obtained deidentified log data for the users’ dietary log, steps, weight, texting records (server logs of messages sent to, and received from, the coach absent any content), and demographic characteristics from Noom. Initially, we reviewed data for 93,814 users from five tables—meal logs, text sent and received log, step logs, weight logs, and user profile—and we extracted and included for analysis data for users who fulfilled the study criteria.

Dietary logs comprised records in which users entered details about consumption time and names of dishes. Weight logs contained manually entered records of their weight. Step logs included passive data automatically collected from the users’ mobile devices. Message logs comprised server records of message transmission when user messages were sent to, or received from, coaches.

In detail, from an individual *i* at a *T_i_* length of univariate time series *l_i_*, we obtained an *i* time series with a heterogeneous length (*l_i_* ∈
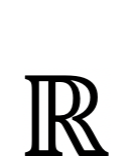
*^Ti^*). For a time series with a length <30 observations, linear interpolation was adopted because previous research suggested that linear interpolation of weight can estimate missing data [20]. As a moving average can make a time series shorter, the time series needs to be elongated in advance. Thus, for the long length of time series, we sampled values with the same interval and obtained the same length for each time series by resampling. In doing so, we were able to alleviate noise in the time series stemming from within-observer variability; for example, when weight was not measured at the same time every day, we smoothed the data after applying a centered moving average with a window size of 15 observations and finally obtained a time series with a length of 16 observations [21].

Exponential moving average methods such as double exponential smoothing or Holter-Winter smoothing were not considered because these methods use the most recent past value, which is often indicative of the near future rather than the remote past. Furthermore, these methods are more appropriate for economic indicators that entail a trend or seasonality in a long-term perspective [22,23]. To reduce time series noise in the distance between 2 time series, rather than considering the long-term trend or slope over time, a simple moving average was adopted, especially considering that this weight loss program was conducted only for a short period of 16 weeks.

Furthermore, we performed mean-variance scaling to normalize the weight range for each user by adjusting the mean of the time series as 0 and the SD as 1. Subsequently, to identify weight loss trajectories, we performed k-means clustering with dynamic time warping (DTW), which is considered the distance between 2 time series. k-means clustering is one of the most commonly used algorithms for partitioning clusters in which each cluster has a centroid (prototype), which reflects the mean value of its objects (Multimedia Appendix 1) [24].

This clustering algorithm minimizes the total distance between all objects in a cluster and the centroid. In this algorithm, we used DTW as the distance between 2 objects to calculate the total sum of distances. DTW is a similarity measure between time series that considers the shape of 2 time series [25,26]. Using DTW, we could cluster time series with similar patterns of change, regardless of the time points of the weight records [24]. As k-means clustering requires researchers to choose the number of clusters (called *k*), the quality of clusters may vary by *k* [27]. To evaluate the quality of clusters, we used elbow methods by inspecting the total sum of distances in all clusters (inertia) through a comparison of the number of clusters from 2 to 10 (Multimedia Appendix 2) [28,29].

### Outcome

Our primary objective is to identify weight loss trajectories over 16 weeks among mobile app users who are overweight. The secondary objective is to determine factors related to behavioral and app use characteristics eliciting weight loss.

We defined both initial and final weights. Initial weight does not refer to the weight the user entered when signing up to use the app. Rather, it represents the first weight entered in the period before coaching and 1 week after the commencement of coaching. This first weight logged is closer to the users’ actual initial weight because they may not have measured their actual weight before entering it during registration. Before coaching, they may have tended to underestimate their weight. Final weight refers to the last weight measured at week 16 (the last program week) or last weight measured at week 17 only if the user did not enter their weight at week 16. We defined final weight in terms of the percentage of weight loss after 16 weeks (stable weight [<2% change], gain of >2%, loss of 2%-5%, loss of 5%-10%, loss of 10%-15%, and loss of >15% compared with initial weight) [30].

### Statistical Analysis

To identify behavioral and app use characteristics in each cluster, we first applied the Kolmogorov-Smirnov test to identify the normality of distribution. According to these results, we performed the Kruskal-Wallis test for normally distributed variables and 1-way ANOVA for normally distributed variables. We used ANOVA for continuous variables to identify whether the means in each k group were equal (*H*_0_:*μ*_1_ = *μ*_2_ , ... , = *μ_K_*) and the Kruskal-Wallis test to determine whether population medians were equal (null hypothesis) [31].

The chi-square test was used to identify whether the distributions of categorical variables in each group were equal. Among the clusters, we chose three (clusters 1, 2, and 3) that showed a converging pattern over 8 weeks and compared the use characteristics of these 3 clusters before and after 8 weeks. To identify differences in app use and behavioral characteristics among these clusters, we compared characteristics by week, from week 1 to week 16, using 2-sample, 2-tailed *t* tests and ANOVA.

For each comparison, effect sizes were calculated depending on the type of variable and number of groups compared to determine the possibility of type 1 statistical error. As the statistical significance was not adequate enough to compare groups of a large population, which will almost always demonstrate a significant difference because of statistical power, we additionally calculated effect sizes referring to the magnitude of group differences as means or proportions [32]. For continuous variables, Cohen *d* was calculated when 2 independent means were compared and eta squared (*η*^2^) was calculated when >2 independent means were compared (ANOVA) [33,34]. For categorical variables, phi (*Φ*) was calculated for a 2×2 contingency table and Cramer V was used for >2 categories [35].

In this study, a 2-sided *P* value of <.05 and an effect size greater than small, depending on its type (*η*^2^≈0.01: small, Cramer V≈0.01: small, and Cohen *d≈*–0.20 to +0.20: small), were considered significant. Statistical analyses were performed with Python (version 3.68; Python Software Foundation) and with the tslearn (version 0.4.1) library [36,37].

### Ethics Approval

This study was approved by the institutional review board at Advarra (Columbia, Maryland, United States; CR00123125). The deidentified nature of the retrospective log data made obtaining informed consent unnecessary.

## Results

### Overview

**Figure 1:**
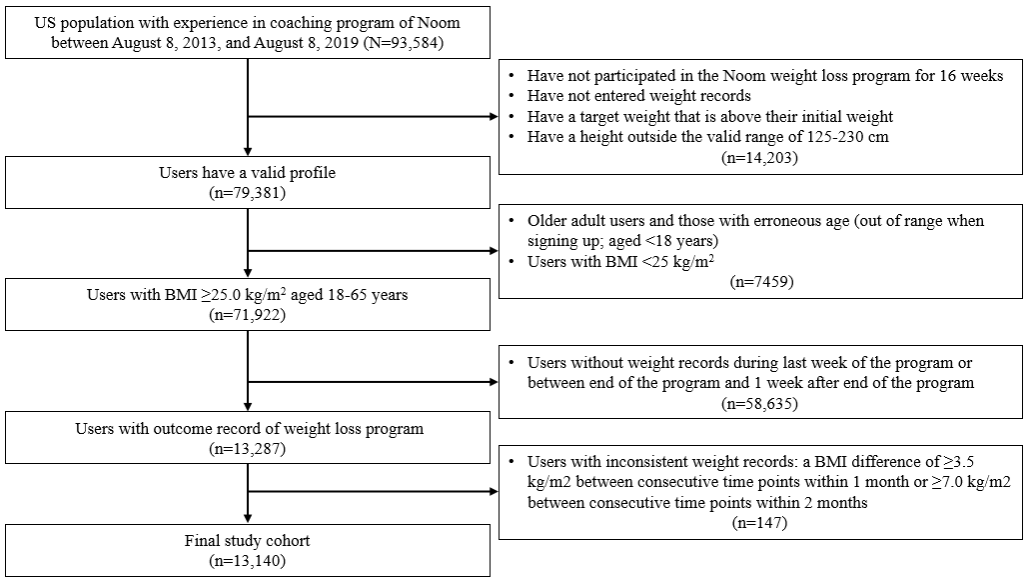
Process of selection of eligible users.

Of the 93,584 unique users who have used Noom, 14,203 (15.18%) were excluded for the following reasons: did not participate in weight loss program, did not enter weight records, target weight more than initial weight, or height out of range. Of the remaining 79,381 users, 7459 (9.40%) were excluded because they were not overweight or they did not meet the age criteria, leaving 71,922 (90.60%) users. Of these 71,922 users, 58,635 (81.53%) were excluded for not completing the program, leaving 13,287 (18.47%) users. Finally, of these 13,287 users, 147 (1.11%) with inconsistent weight records were excluded, leaving 13,140 (98.89%) users who had exhibited adherence to the app (Figure 1).

### Baseline Characteristics

At baseline, the proportion of female users (12,093/13,140, 92.03%; Table 1) was larger than that of male users (1047/13,140, 7.9%). The average age of the users was 43.9 (SD 10.9) years, and the average height was 166.4 (SD 7.4) cm. Overall, the mean initial BMI was 33.6 (SD 5.9) kg/m^2^, which decreased to 31.6 (SD 5.7) kg/m^2^ on average.

**Table 1:** Baseline characteristics (N=13,140).

Variables		Values
**Sex, n (%)**		
	Female	12,093 (92.03)
	Male	1047 (7.9)
Age (years), mean (SD)		43.9 (10.9)
Height (cm), mean (SD)		166.4 (7.4)
Initial weight (kg), mean (SD)		93.2 (18.1)
Initial BMI (kg/m^2^), mean (SD)		33.6 (5.9)
Final weight (kg), mean (SD)		87.6 (17.2)
Final BMI (kg/m^2^), mean (SD)		31.6 (5.7)
Weight loss (kg), mean (SD)		–5.7 (5.5)
BMI loss (kg/m^2^), mean (SD)		–2.0 (1.9)

### Weight Loss Trajectories

**Figure 2:**
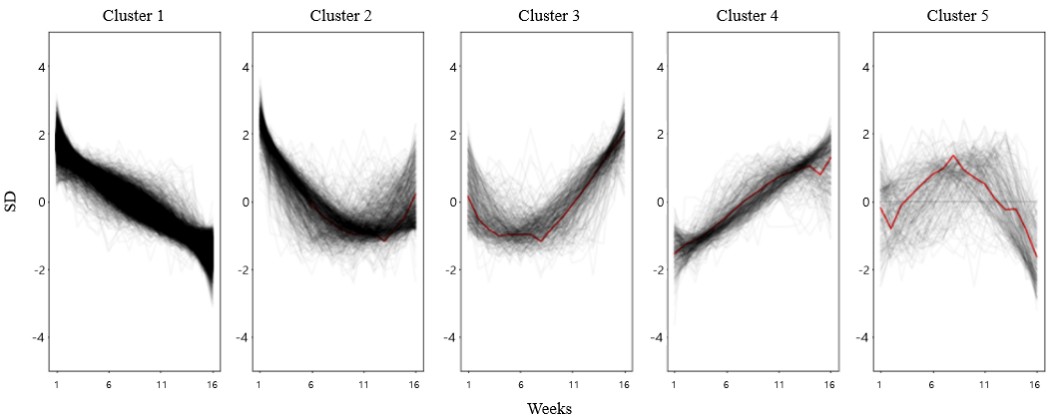
Clustered weight loss trajectories using k-means with dynamic time warping. Each black line signifies an individual user’s weight loss journey. The red line represents the weight loss trajectory of each cluster.

We explored the optimal number of clusters (k) by changing the number of clusters from 2 to 10. Using elbow methods with visualization, we determined the optimal number of clusters (k) to be 5 (Multimedia Appendix 2) for clustering users with adherence. Each user was assigned to a cluster: 85.96% (11,295/13,140) of the users were assigned to cluster 1, followed by 6.34% (833/13,140) assigned to cluster 2. Users in cluster 1 (sharp decrease) exhibited a decrease in their weight without plateaus. Users in cluster 2 (moderate decrease) initially showed a sharp reduction in weight, after which the slope of weight loss plateaued. Users in cluster 3 (yo-yo) exhibited a decrease in weight, but in the middle of the program, they exhibited a gain in weight. Although cluster 2 (moderate decrease) and cluster 3 (yo-yo) exhibited convergence in weight loss patterns for the initial 8 weeks, users in cluster 2 maintained their weight, whereas those in cluster 3 gained weight after 8 weeks. Users in cluster 4 (stable or increase) gained weight, and those in cluster 5 (other) did not show a convergence pattern because of the partitioned-clustering approach (Figure 2).

There were no significant differences in medians of the initial BMIs of the clusters (*η*^2^<0.001; *P*=.43). However, there was a significant difference in weight reduction class (Cramer V=0.241; *P*<.001). In cluster 1 (sharp decrease), of the 11,295 users, 4541 (40.20%) exhibited weight loss of 5%-10%, which was the largest proportion among the clusters. In addition, this cluster comprised the largest proportion of users recording weight loss ranging from 10% to 15% (2206/11,295, 19.53%). In cluster 2 (moderate decrease), of the 833 users, 281 (33.7%) exhibited weight loss of 2%-5% (Table 2).

Furthermore, the 5 clusters showed different use characteristics in terms of frequency of meal and weight record adherence (*η*^2^=0.056; *P*<.001, and *η*^2^=0.024; *P*<.001, respectively). Cluster 1 users recorded their meals and weight most frequently (median 18.5, IQR 4.1 times per week and median 4.9, IQR 1.8 times per week, respectively), followed by cluster 2 users for meal record adherence (median 16.1, IQR 5.2 times per week). Cluster 2 users entered their weight a median of 4.4 (IQR 1.8) times per week.

The median number of sent and received messages among the clusters did not show a significant difference (*η*^2^<0.001; *P*=.12, and *η*^2^=0.001; *P*=.001, respectively). However, there was a significant difference in the median number of steps (*η*^2^=0.002; *P*<.001).

**Table 2:** Comparison of app use and behavioral characteristics among the 5 clusters (N=13,140).

Variables		Clusters						*P* value		Effect size^a^
		Cluster 1 (sharp decrease), n=11,295	Cluster 2 (moderate decrease), n=833	Cluster 3 (yo-yo), n=384	Cluster 4 (stable or increase), n=401	Cluster 5 (other), n=227				
Initial BMI (kg/m^2^), median (IQR)		32.5 (7.7)	32.6 (7.5)	32.6 (6.7)	33.5 (7.5)	32.0 (7.4)	.43		<0.001	
**Weight loss class, n (%)**								<.001		0.241
	Gained >2%	438 (3.87)	96 (11.52)	94 (24.48)	124 (30.92)	25 (11.01)				
	Stable	1107 (9.80)	331 (39.74)	205 (53.39)	195 (48.63)	102 (44.93)				
	Lost 2%-5%	2454 (21.73)	281 (33.73)	62 (16.15)	52 (12.97)	72 (31.72)				
	Lost 10%-15%	2206 (19.53)	19 (2.28)	10 (2.60)	6 (1.50)	4 (1.76)				
	Lost >15%	549 (4.86)	8 (0.96)	0 (0)	8 (2)	2 (0.88)				
Meal record adherence (records per week), median (IQR)		18.5 (4.1)	16.1 (5.2)	15.1 (6.2)	15.5 (6.1)	16.2 (6.7)	<.001		0.056	
Weight record adherence (n per week), median (IQR)		4.9 (1.8)	4.4 (1.8)	4.0 (2.0)	4.162 (2.3)	3.6 (2.7)	<.001		0.024	
Sent messages (n per week), median (IQR)		2.1 (1.6)	2.1 (1.6)	1.9 (1.4)	1.9 (1.7)	1.9 (1.8)	.12		<0.001	
Received messages (n per week), median (IQR)		3.0 (1.7)	3.0 (1.6)	2.8 (1.5)	2.8 (1.925)	2.8 (1.954)	.001		0.001	
Steps (per day), median (IQR)		5469.2 (4236.7)	5190.6 (3979.2)	5101.5 (3943.0)	5070.0 (3944.5)	4809.6 (3974.8)	<.001		0.002	

^a^Effect size was calculated using eta squared (*η*^2^) for continuous variables and Cramer V for categorical variables (*η*^2^≈0.01: small, *η*^2^≈0.09: moderate, and *η*^2^≈0.25: large; Cramer V≈0.01: small, Cramer V≈0.30: moderate, and Cramer V≈0.50: large).

### Weight Regain and Steady Loss

**Figure 3:**
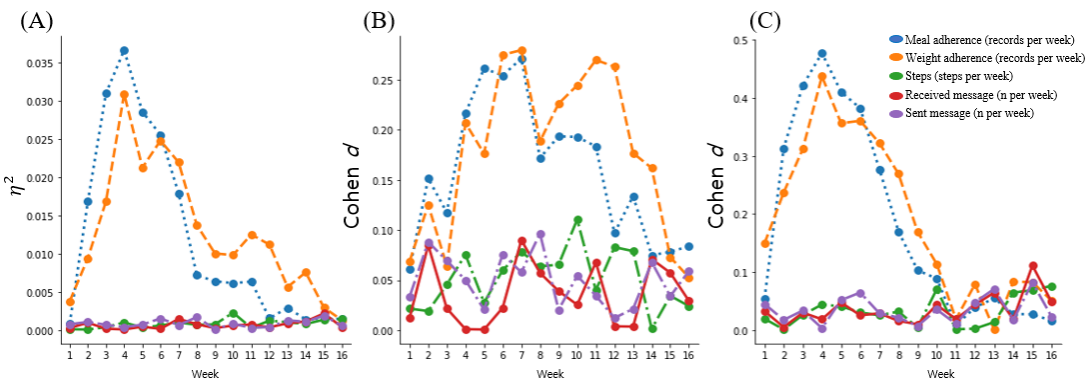
Comparison of longitudinal app use and behavioral characteristics for individual clusters. (A) Eta squared from analysis of variance test with clusters 1, 2, and 3. (B) Cohen *d* from *t* test between clusters 1 and 3. (C) Cohen *d* from *t* test between clusters 2 and 3 (η^2^≈0.01: small, η^2^≈0.09: moderate, and η^2^≈0.25: large; Cohen *d*≈–0.20 to +0.20: small, Cohen *d*≈0.50: moderate, and Cohen *d*≈0.80: large).

By the middle of the 16-week program, clusters 1, 2, and 3 showed similar weight loss slopes. However, after this point, the trajectory of cluster 3 showed a rebound. Therefore, we further compared the characteristics of these clusters. Longitudinal app use and behavioral characteristics of clusters 1, 2, and 3, including weight record, meal record, and steps, as well as messages sent and received, were plotted over 16 weeks (Figure 3). Overall, although no differences were observed in both meal and weight record adherence among the clusters in the first week, a significant difference peaked within the initial 8 weeks (Figures 3A, 3B, and 3C). The Cohen *d* values of clusters 1 and 2 were maintained between 0.17 and 0.28 even after 8 weeks, and there was a drastic decrease after 15 weeks; however, those of clusters 2 and 3 peaked at 4 weeks and decreased thereafter. Among these clusters, adherence peaked between 2 and 4 weeks and then showed divergence on average, with the highest adherence maintained in the clusters 1, 2, and 3, in that order (Multimedia Appendix 3). In contrast, there were no significant differences in average daily steps or messages sent and received for the entire 16-week period.

## Discussion

### Principal Findings

In this retrospective study, we examined the weight loss trajectories of a large population of individuals who completed a mobile app intervention program. Using a machine learning approach and applying a clustering method and shape-based time series similarity, we found 5 primary clusters. By comparing the use characteristics of each cluster, we found that meal and weight record adherence affected weight trajectory more than the sent and received messages and daily steps.

To evaluate the validity of the clusters, we assessed both cross-sectional and longitudinal differences in use characteristics indicative of cluster attributes, as well as basic clustering validity indices such as inertia. In doing so, we noted that the magnitude of self-monitoring–related app use, such as adherence to recording one’s meals and weight, depended more on clustering membership, especially between individuals with a steady weight loss and those who initially lost weight but later regained it. Longitudinally, app use between steadily losing or regaining weight differed greatly after initial use. During the first week, almost all users showed high adherence to the app; however, divergence in adherence began to appear from week 2. Cohen *d* values for meal and weight adherence between cluster 2 (moderate decrease) and cluster 3 (yo-yo) peaked at week 4 and gradually decreased until the end of the program. Although clusters 2 and 3 similarly showed high adherence to recording meal and weight until week 3, adherence decreased at the end of the program (Multimedia Appendix 3): adherence decreased more steeply in cluster 3 than in cluster 2. From these results, we discerned that cluster membership depended largely on how users maintained app and program adherence, reflecting a willingness to perform self-monitoring, from week 3 onward.

Unlike studies that used a variable-centered approach, which assumes that a set of *average* parameters can be estimated for all individuals drawn from a population, this study was based on a *person-centered approach*, categorizing individuals into common subpopulations and determining whether subgroups of similar participants exist [38]. Although our method did not assume any parameters, we found 5 subpopulations that showed differences in use patterns. Our method is consistent with previous research demonstrating that it is necessary to divide an entire population into subgroups and then perform more detailed analysis, rather than to analyze the entire population as a single group [14,39,40]. Considering the well-recognized heterogeneity of weight loss outcomes and variability of outcomes in the intervention period, these results lay the foundation for the design of an obesity management program using mobile phones [41].

### Identification of Weight Loss Trajectories

Previously, to discover patterns of weight loss, research has applied statistical clustering methods, principal component analysis, and latent class analysis. In doing so, three primary weight loss patterns over time have been proposed (modest loss, moderate loss, and substantial loss) [14,42-44]. Most of these analyses were based on on-site interventions, although some studies focused on weight loss trajectories using samples from clinical trials. As there might be gaps in the efficacy of interventions between a clinical trial setting and a real-world setting [45], our results, which were obtained using a large data set, could be considered to broadly reflect a real-world context [46]. Thus, our study expounds on previous evidence of mobile-based interventions by considering the shape, and calculating the similarity, of time series with DTW and by comparing the behavioral characteristics of individual app users.

The methods used in this study can be adopted in research related to long-term weight loss or maintenance. It is known that it can be difficult to keep weight off because of various reasons, such as having an obesogenic environment or difficulty in managing physiological responses to weight loss [47]. In addition, hurdles such as the occurrence of a weight loss plateau appear during long-term maintenance. For long-term weight management, it may be possible to identify long-term weight loss trajectories using our method as a patient-centered approach. Although some studies have attempted to identify long-term weight loss trajectories in on-site management [48,49], no study has examined long-term weight trajectories using nonparametric analyses. Further research identifying weight loss plateaus or rebounding trajectories in the long term could reveal factors contributing to, or mitigating, long-term weight loss.

### Application of Weight Loss Trajectories

Obesity management using mobile health technology enables the design of just-in-time adaptive interventions for behavioral support that directly correspond to needs determined by using real-time data collected from user records [50]. Given the use of time series clustering at each week, our method was able to infer membership in a time series despite the different number of observations on each device. Using these data on membership at each time point, clinicians or counselors can estimate what a user’s weight trajectory might look like. Accordingly, by providing feedback, they can support the user to self-monitor their weight timely.

Nevertheless, when the scale of 2 time series shows a large difference (eg, weight time series of users weighing 170 kg vs those weighing 70 kg), DTW, the distance between 2 time series, may be increased. To measure only the shape of a time series and not actual weight values, our method reflects factors that may be contributing to weight loss by rescaling. In this study, we comprehensively clustered weight loss patterns among participants who needed to lose weight (ie, those in the preobese or higher category) by adjusting the weight scale.

In addition, research has shown that weight loss trajectories can be related to clinical indicators associated with comorbidity, wherein weight loss patterns corresponded with improvements in blood pressure, triglyceride, and blood glucose levels [49]. Similarly, our approach provides added clinical meaning beyond mere weight loss. In addition, researchers have demonstrated that it is possible to predict the amount of weight a user will lose at the end of a program using interpretable artificial intelligence (AI), which can be used in the coaching process [51]. With explainable AI–used adherence as a feature, adherence becomes a contributing factor to weight loss. Furthermore, the membership of a cluster is interpreted as a contributing factor; for example, membership of cluster 3 (yo-yo) contributed to weight loss, but users in this cluster gained weight after 8 weeks. Interpreting the AI results, coaches can consult with users about their current app use, trajectory, and predicted weight loss, allowing them to intervene early in a program to improve otherwise suboptimal outcomes.

### Use Factors Related With Weight Loss

In this study, although individuals were not classified by variables related to self-monitoring, the results revealed that two variables (meal and weight record adherence) showed an association with weight loss after clustering. Among behavioral strategies, self-monitoring is recommended for maintaining lost weight, even when using mobile apps [19,52,53]. It has been posited that self-monitoring serves as the initial step in a feedback loop that includes observation and recording, self-evaluation, and self-reinforcement, which can help individuals decide to adjust behaviors [54]. Individuals with a high meal adherence may review their dietary behavior more frequently and arrive at opportunities for self-evaluation and self-reinforcement.

Although we did not explicitly show that weight loss of 5% to 10% would be suitable as a cutoff for responders, our findings indicated that among individuals who used the app, those who adhered to the app more regularly lost more weight, which could be regarded as a weight loss response. Nevertheless, our findings also showed that the weight trajectory can rise again if compliance is not maintained, despite being high initially. Sufficient evidence suggests that early weight loss can be a predictive factor for long-term weight loss [55-58]. Similarly, our results showed that an early use pattern can also be a predictive factor for weight loss outcomes in a 16-week program. This study compared a group that lost and regained weight with a group that exhibited a moderate decrease in weight. The 2 groups showed a difference in app adherence after only 2 weeks, and this difference increased continuously for 8 weeks. In line with our results, a previous study also showed that early intervention affects short- and long-term weight loss in a weight loss program, although it did not examine web-based or mobile programs [59].

### Limitations

Our study includes some inherent limitations because of its selection of participants and the retention rate, which could pose a selection bias. First, the participants in this study had purchased a subscription to a weight loss program. As our sampled participants may have had a higher intention to lose weight than individuals who use free web-based interventions, the former may have exhibited a relatively higher retention rate than the latter. The average 30-day retention rate for health and fitness apps in the United States is 3.4% and that for mental health apps is 3%-8% [60-62]. Considering these values, some selection bias may have occurred in this study.

Second, this work may have introduced latent sampling bias through the process of participant selection because of the criterion by which we included only users with weight records during the last week of the program or between the end of the program and 1 week after the end of the program. Although it was necessary to include this criterion to identify the final outcome of the weight loss program, it may have led to the inclusion of users who may have been more compliant and more likely to be successful with weight loss. Therefore, our findings need to be interpreted considering these limitations. In addition, our participants were selected from users who entered their records as a minimal requirement, which could affect the interpretation and generalization of our findings. For example, because some of the users who did not enter their records showed nonadherence, the factors related to use patterns may not predict their weight trajectories. Our results may be reliable for users who have at least minimal adherence. If the inclusion criteria were stricter, such as recording all 3 meals 1 day per week or recording 1 meal per day per week, approximately 3000 or 15,000, respectively, of the participants would have been additionally excluded. Therefore, we set inclusion criteria that required 1 recording of a meal per week as a condition for minimal self-monitoring.

In addition, our participants consisted of 92.03% (12,093/13,140) women and 7.9% (1047/13,140) men. Although it can be thought that there may be a bias in the selection process of the participants, with a high proportion of women, statistics for participants in previous studies of mobile weight loss apps showed high proportions of women [63-65]. Although there are some magnitudes of difference in the proportion of women among the participants, our may be adopted in that the statistics of participants in previous studies were consistent with ours.

In addition, we conducted an experiment to identify whether a latent selection bias was present in the exclusion of participants with inconsistent weight in 2 consecutive records (Multimedia Appendix 4). Despite including these participants, the number of optimal clusters remained at 5, and each cluster showed the same 5 shapes presented in this paper. Although the membership of each cluster differed (clusters with participants with erroneous records comprised 11,184, 955, 363, 575, and 245 users, respectively, for clusters 1 to 5, whereas those excluding these participants comprised 11,295, 833, 384, 401, and 277 users, respectively), the comparative analysis showed consistent results, wherein (1) the mean initial BMI did not differ significantly; (2) after the program, cluster 1 users exhibited the highest weight loss; and (3) weight and meal record adherence was relatively higher in clusters 1 and 2 than in the other clusters.

In terms of data reliability, although we accounted for noise in the weight loss time series, it was difficult to determine whether each user had correctly entered meal skipping and weight (data reliability stems from user entries).

Finally, although sustainable weight loss in the long term is important in obesity management, we did not explore long-term outcomes because of missing values from the users’ logs. Thus, a long-term cohort study is needed to obtain more reliable evidence on long-term outcomes. Despite these limitations, our work provides evidence indicating that weight loss trajectories depend on overall adherence and early adherence to self-monitoring in a limited observation period (16 weeks).

### Conclusions

Using time series clustering, we identified 5 distinct profiles of weight change over a 16-week weight management intervention through a mobile app. We found that overall adherence and early adherence to self-monitoring could be predictive factors for greater weight loss success.

## Appendix

Multimedia Appendix 1 Pseudocode for weight loss trajectories: k-means clustering with dynamic time warping.

Multimedia Appendix 2 Sum of distance with reference to number of clusters (excluding users with inconsistent weight records).

Multimedia Appendix 3 Boxplot of the use factors by week.

Multimedia Appendix 4 Robustness of clustering with modified selection flow.

## Abbreviations

AI: artificial intelligence
DTW: dynamic time warping
