# Supplementary material for: Weight Loss Trajectories and Related Factors in a 16-Week Mobile Obesity Intervention Program: Retrospective Observational Study
Source: J Med Internet Res. 2022 Apr 15;24(4):e29380. doi: 10.2196/29380 (PMC9055473; doi:10.2196/29380)
Supplement: Multimedia Appendix 1 [file jmir_v24i4e29380_app1.docx]

Weight loss trajectories and related factors in a 16-week mobile obesity intervention: A retrospective observational study

Ho Heon Kim^1^ RN; Young In Kim^1,2^ MD; Andreas Michaelides^2^ PhD; Yu Rang Park^1^,* PhD

Supplement 1. Pseudocode for weight loss trajectories: k-means clustering with dynamic time warping (DTW)

| **Algorithm 1:** k-means clustering with dynamic time warping (DTW) | |
| --- | --- |
| **Input**: | |
| $\mathcal{X=\{}l_{1},\ldots,$ $l_{i}\}$ : univariate time series with each length $T_{i}$  *K*: number of desired clusters  T: max number of allowed iteration  R: Sampling rate  N: Window size for moving average | |
| **Output:** | |
| $\left\{ m_{1}^{(t)}, \ldots,m_{k}^{(t)} \right\}$: set of membership in each *k* cluster | |
| **Procedure:** | |
| 1: | **For all** $l_{i}$ in $\mathcal{X}$ **do** |
| 2: | $\bar{l}_{i}=$Resampling($l_{i}$ ,R) |
| 3: | $\tilde{l}_{i}$ = Centered SMA ($\bar{l}_{i}$, N) |
| 4: | $\left\Vert l_{i} \right\Vert$ = Mean-Variance scaling($\tilde{l}_{i}$) |
| 5: | $\bar{\mathcal{X}}=\{\left\Vert l_{1} \right\Vert, \ldots, \left\Vert l_{i} \right\Vert\}$ |
| 6: | **Do** k-means clustering with DTW as distance |
| 7: | $\boldsymbol{d}_{\boldsymbol{i,}\boldsymbol{c}_{\boldsymbol{k}}^{\boldsymbol{(t)}}}$ **=** DTW between *i* time series and each centroid $c_{k}^{(t)}$ in *t*-th iteration |
| 8: | t = 0 |
| 9: | $C^{(t)}=\left\{ c_{1}^{(t)}, \ldots,c_{k}^{(t)} \right\}$ // Randomly choose *k* initial centroids among $\left\Vert l_{i} \right\Vert$ |
| 10: | **While** t $\leq$ T or $\mathcal{L}^{(t)}> \mathcal{L}^{(t-1)}$ |
| 11: | t += 1 |
| 12: | **For** j = 1, ..., *i* **do** |
| 13: | $m_{k,j}^{(t)}=\left\Vert l_{j} \right\Vert, if k= argmin_{k}(d_{j,c_{k}})$ |
| 14: | **For** *k* = 1,…, *K* **do:** |
| 15: | $m_{k}^{(t)}=${$m_{k,j}^{(t)} for j in 1,\ldots,i\}$ // cluster membership |
| 16: | $C^{(t)}=\{c_{k}^{(t)}\boldsymbol{=}\frac{\boldsymbol{1}}{\boldsymbol{\vert}\boldsymbol{c}_{\boldsymbol{k}}^{\boldsymbol{(t)}}\boldsymbol{\vert}}\sum_{\left\Vert l_{i} \right\Vert\in m_{k}^{(t)}} \left\Vert l_{i} \right\Vert for all k=1,\ldots,K\}$ // Update centroid |
| 17: | $\mathcal{L}^{(t)}$ = $\sum_{k=1}^{K} \sum_{\left\Vert l_{i} \right\Vert\in m_{k}}^{I} d_{i,c_{k}^{(t)}}$ |
| 18: | **return** $\left\{ m_{1}^{(t)}, \ldots,m_{k}^{(t)} \right\}$ |
