# Supplementary material for: Weight Loss Trajectories and Related Factors in a 16-Week Mobile Obesity Intervention Program: Retrospective Observational Study
Source: J Med Internet Res. 2022 Apr 15;24(4):e29380. doi: 10.2196/29380 (PMC9055473; doi:10.2196/29380)

Weight loss trajectories and related factors in a 16-week mobile obesity intervention: A retrospective observational study

Ho Heon Kim^1^ RN; Young In Kim^1,2^ MD; Andreas Michaelides^2^ PhD; Yu Rang Park^1^,* PhD

Supplementary 2. Sum of distance with reference to number of clusters (excluding users with inconsistent weight records).


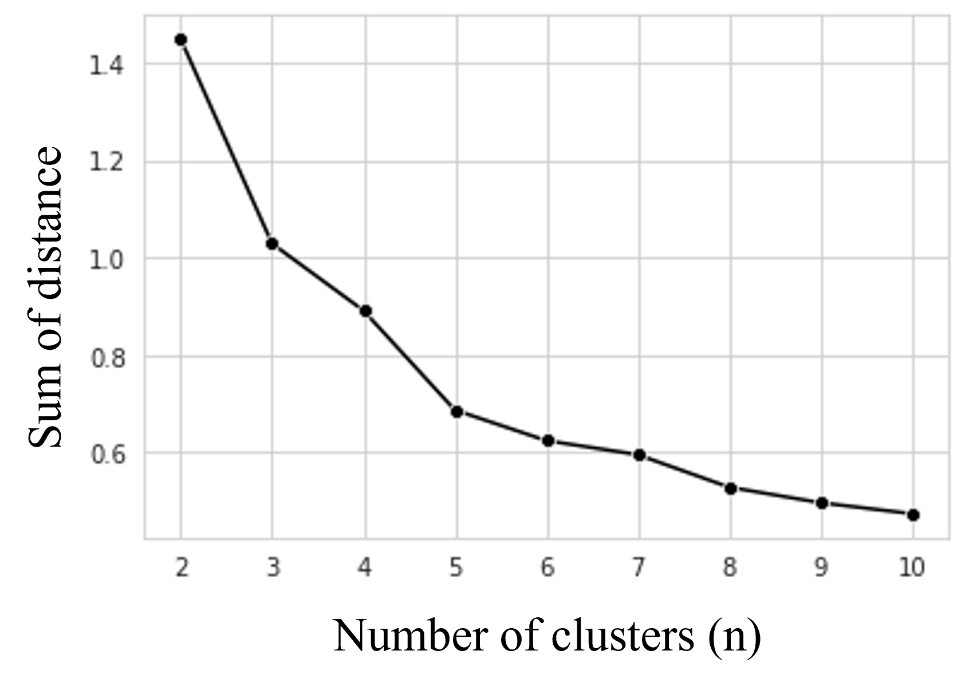

Supplement: Multimedia Appendix 2 [file jmir_v24i4e29380_app2.docx]
