# Supplementary material for: Weight Loss Trajectories and Related Factors in a 16-Week Mobile Obesity Intervention Program: Retrospective Observational Study
Source: J Med Internet Res. 2022 Apr 15;24(4):e29380. doi: 10.2196/29380 (PMC9055473; doi:10.2196/29380)
Supplement: Multimedia Appendix 4 [file jmir_v24i4e29380_app4.docx]

Weight loss trajectories and related factors in a 16-week mobile obesity intervention: A retrospective observational study


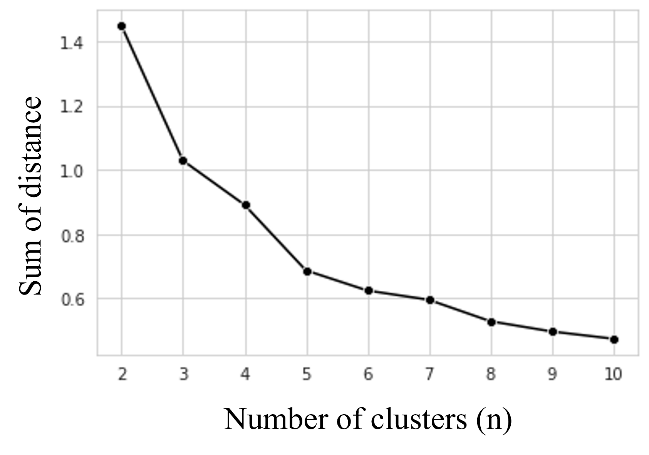
Ho Heon Kim, Young In Kim, Andreas Michaelides, Yu Rang Park

Multimedia appendix 4-1. Sum of distance with reference to number of clusters (excluding users with inconsistent weight records).


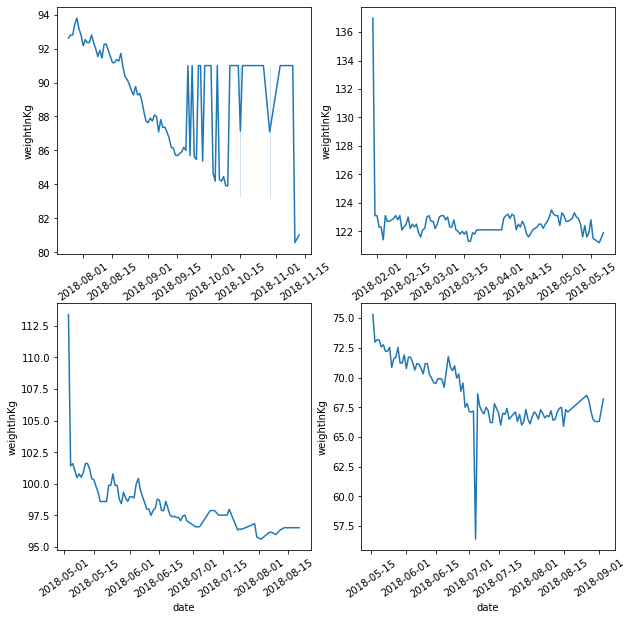

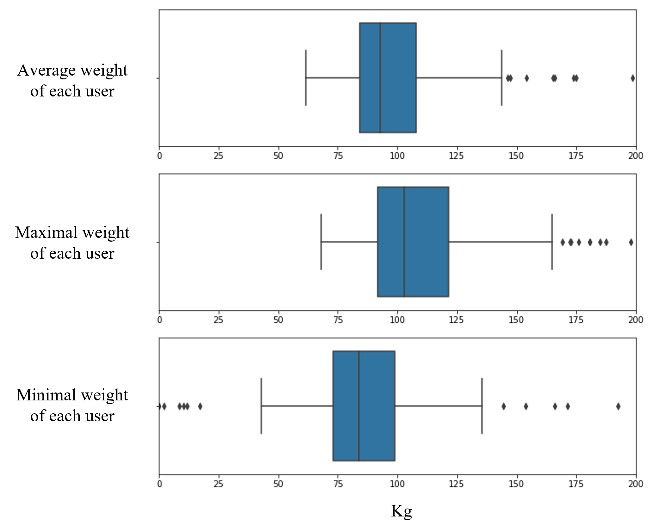


Multimedia appendix 4-2. Distribution of the weight of excluded users’ with inconsistent weight records; left: users with a BMI difference of ≥3.5 kg/m^2^ between consecutive time points, right: examples of excluded users’ weight log.


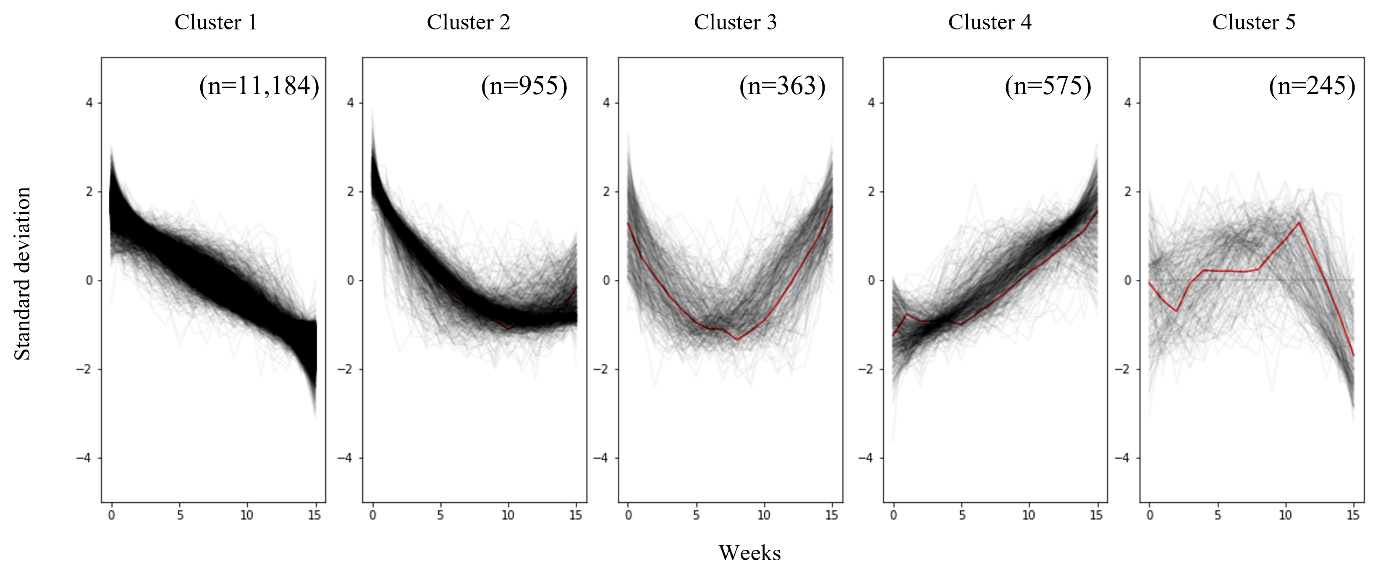


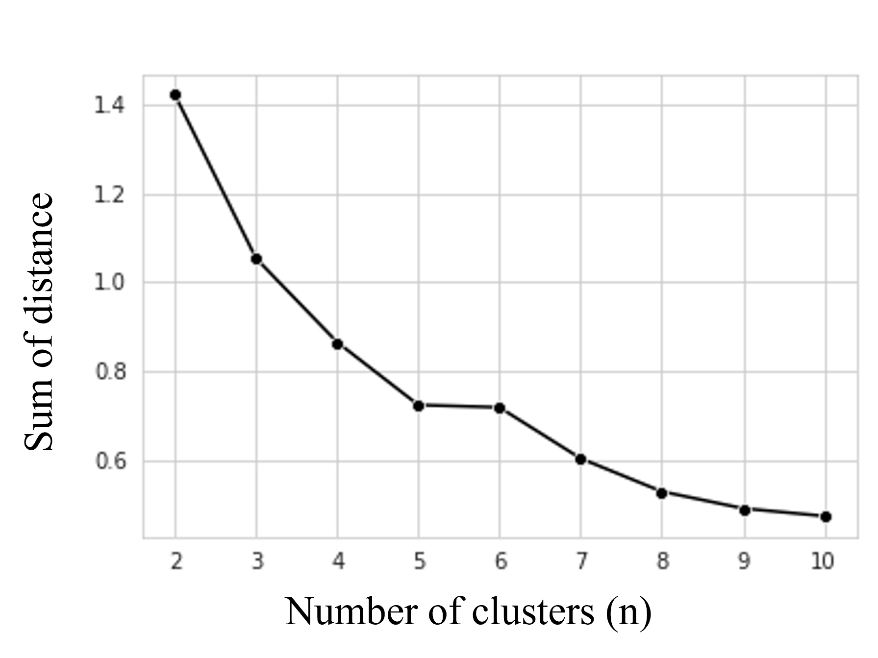
Multimedia appendix 4-3. A: Clustered weight loss trajectories including participants with inconsistent weight records (users with a BMI difference of ≥3.5 kg/m^2^ between consecutive time points); B: Sum of distance with reference to number of clusters.
